# Empathic behavior according to the state of others in mice

**DOI:** 10.1002/brb3.986

**Published:** 2018-05-29

**Authors:** Hiroshi Ueno, Shunsuke Suemitsu, Shinji Murakami, Naoya Kitamura, Kenta Wani, Motoi Okamoto, Yosuke Matsumoto, Shozo Aoki, Takeshi Ishihara

**Affiliations:** ^1^ Department of Medical Technology Kawasaki University of Medical Welfare Okayama Japan; ^2^ Department of Medical Technology Graduate School of Health Sciences Okayama University Okayama Japan; ^3^ Department of Psychiatry Kawasaki Medical School Okayama Japan; ^4^ Department of Neuropsychiatry Graduate School of Medicine Dentistry and Pharmaceutical Sciences Okayama University Okayama Japan

**Keywords:** behavior, distress, empathy, mouse, pain, social behavior

## Abstract

**Introduction:**

Empathic behavior is essential for social activities in social animals. Therefore, lack of empathy is a feature of several neuropsychiatric disorders. However, the underlying mechanisms of empathy and which animals possess it remain unclear. In this study, we investigated whether mice show empathic behavior.

**Methods:**

We tested mice for empathy‐like behaviors toward conspecifics who were distressed. We investigated behavioral changes in cage‐mate or stranger mice.

**Results:**

When the conspecific mice were in a painful state, subject mice showed preferential approach behavior toward them, presumably recognizing the state. Both visual information and olfactory information are indispensable for this empathic behavior.

**Conclusions:**

These results suggest that the mouse recognizes the emotional state of the conspecific and engages in social interaction. The results of this study are useful for the elucidation of the causal mechanisms involved in neuropsychiatric disorders and may contribute in the development of novel treatment targets.

## 1 INTRODUCTION

Impaired empathy is a characteristic of various neuropsychiatric disorders such as autism spectrum disorder (ASD) and schizophrenia (Bernhardt & Singer, 2012; Bird et al., 2010; Bora, Yucel, & Pantelis, 2009). It is important to infer others’ emotional state for smooth communication. In recent years, image research with human functional magnetic resonance imaging (fMRI) has shown that the anterior cingulate cortex is involved in empathy (Danziger, Faillenot, & Peyron, 2009; Singer et al., 2004). However, the underlying mechanisms involved in empathy have not been elucidated, while their clarification would be useful in elucidating the causes of neuropsychiatric disorders. In this study, we investigated whether mice, frequently used as laboratory animals, show empathic behavior.

Empathy is the ability to understand what other individuals feel and to share that feeling (Fuchsman, 2015). Empathy is important for social animals (Panksepp & Lahvis, 2011). Historically, empathy has been thought to be a high‐level cognitive process. However, Darwin already indicated numerous examples of empathy and sympathy in animal species (Darwin, 1879, 1890). In recent years, empathic and sympathetic behaviors have been reported in nonhuman primates (de Waal, 2008, 2012, 2013) and rodents (Keum & Shin, 2016; Langford et al., 2006; Li et al., 2014; Sivaselvachandran, Acland, Abdallah, & Martin, in press); it is therefore becoming clear that many nonhuman animals also have empathy. As many animal experiments in recent years have proven, empathy is a biological process (Chen, 2018; Grenier & Lüthi, 2010). That is, both empathy and sympathy in humans have evolved (Decety, Norman, Berntson, & Cacioppo, 2012).

Prosocial behavior signifies action intended to help others without expecting external compensation (Mussen & Eisenberg‐Berg, 1977). Empathy involves the transmission of emotions preceding prosocial behavior. It has been shown that rodents engage in behavior intended to help conspecifics in a distressed state based on empathetic motives (Bartal, Decety, & Mason, 2011).

In order to observe empathy‐like behavior in mice, we investigated for the first time whether there was preference toward conspecifics who were in distress. We examined whether cage‐mate and stranger mice would recognize the state of conspecifics and engage in empathic behavior; recognizing that a conspecific is in distress is important for avoiding harm and providing assistance.

Three types of mice were utilized in this study: tail‐pinched, formalin‐injected, and anesthetized mice. We examined whether test mice would show social preference toward these treated mice compared to control mice. The tail‐pinch method has been used to investigate pain stimulation responses caused by mechanical noxious stimuli (Levine & Morley, 1982; Simone & Bodnar, 1983). The formalin test is used to cause inflammatory pain by injecting formalin into the hind limbs of mice (Dubuisson & Dennis, 1977). As these pain‐testing methods are visually evaluated by a human observer, the test mice in this study could also reliably determine that the treated mouse was in a pain state using visual cues.

The social preference of the mouse can be directly observed by measuring approach or avoidance behavior. To examine social preference in this study, we employed a well‐established experimental protocol (Crawley, 1999, 2000, 2004) where a test mouse is given the choice to approach or avoid a treated mouse, which is confined in a wire cage.

The emotional transmission of pain between mice has been reported in many studies, but its transmission mechanism remains unclear. Clarifying these issues may lead to the elucidation of the mechanism by which humans share emotions, leading in turn to the development of more efficacious treatments for neuropsychiatric disorders.

## 2 METHODS

### 2.1 Animals

All animal experiments were performed in accordance with the U.S. National Institutes of Health (NIH) Guide for the Care and Use of Laboratory Animals (NIH Publication No. 80‐23, revised in 1996) and approved by the Committee for Animal Experiments at Kawasaki Medical School Advanced Research Center. All efforts were made to minimize the number of animals used and their suffering. Eight‐week‐old C57BL/6N male mice were purchased from Charles River Laboratories (Kanagawa, Japan) and housed in cages (five animals per cage) with food and water provided ad libitum under a 12‐hr light/dark cycle at 23°C–26°C. The animals were 11 weeks old at the start of the testing. All behavioral tests were conducted in behavioral testing rooms between 08.00 and 18.00 hr, during the light phase of the circadian cycle. After the tests, all apparatuses were cleaned with 70% ethanol and super hypochlorous water to prevent bias based on olfactory cues. The apparatuses were cleaned after each phase of the present test. Behavioral tests were performed according to the test order described below.

### 2.2 Behavioral procedure

The apparatus consisted of a rectangular, three‐chambered box. Each chamber was 20 × 60 × 40 cm, and the dividing walls were made of clear Plexiglas with small square openings (5 × 3 cm) allowing access into each chamber. Each mouse was placed in the box for 10 min and allowed to freely explore for habituation. This experiment was arranged and conducted using the method of the three‐chambered social approach test (Moy et al., 2004).

In the preference test with cage‐mate mice, a treated or control cage‐mate mouse was placed into one of the wire cages (7.5 × 7.5 × 10 cm; vertical bars, 0.5 cm apart) that were located in the corners of each lateral compartment. The wire cage allowed nose contact between the bars, but prevented fighting. Two mice were placed in opposing wire cages: one intact and one that received one of the following treatments. (1) The animals were deeply anesthetized with a high dose of sodium pentobarbital (50 mg/kg, i.p.). (2) Formalin was injected (50 μl of 4% formalin, diluted in saline) into the dorsal surface of the right hind paw; in this condition, the experiment took place within 40 min. (3) Saline was injected (50 μl of saline) into the dorsal surface of the right hind paw. (4) The animals were tied with plastic clothespins (4 cm) 2.5 cm proximal from the tip of the tail. The clothespin was fixed outside the wire cage. The subject mouse thus had a choice between the intact mouse and the treated mouse. The amount of time spent in each chamber during the second 10‐min session was measured. Data were recorded on video and analyzed using video‐tracking software (TopScan; CleverSys Inc., Reston, VA).

The same method was used in the preference test with stranger mice, which were not housed in the same cage with the subject mouse. This experiment was conducted using two unfamiliar mice (stranger mouse) that had no previous contact with the subject mouse. The subject mouse was placed in the middle chamber and was allowed to explore the three chambers for 10 min. Data were recorded on video and analyzed using video‐tracking software (TopScan).

In the opaque preference test with cage‐mate mice, a cage‐mate mouse was placed into one of two opaque cylinders (25 cm high × 8 cm diameter) that were located in the corners of each lateral compartment. The method was similar to the one used in the preference test with cage‐mate mice. Another cage‐mate mouse received a formalin injection (50 μl of 4% formalin, diluted in saline) and was placed into another of the cylinders. The subject mouse was then placed in the middle chamber and allowed to explore the three chambers for 10 min. Data were recorded on video and analyzed using video‐tracking software (TopScan).

In the odor test, absorbent cotton, which was placed in both opaque cylinders during the previous test, was placed into one of the wire cages that were located in the corners of each lateral compartment. The method was similar to the one used in the preference test with cage‐mate mice. The subject mouse was then placed in the middle chamber and allowed to explore the three chambers for 5 min. Data were recorded on video and analyzed using video‐tracking software (TopScan).

### 2.3 Statistical analysis of behavioral tests

Statistical analysis was conducted using the SPSS software (IBM Corp, Tokyo, Japan). Data were analyzed with two‐tailed *t* tests or two‐way factorial analysis of variance. A *p* value <.05 was regarded as statistically significant. All the data are presented as mean ± *SEM*.

## 3 RESULTS

### 3.1 Degree of interest in anesthetized mice

**Figure 1:**
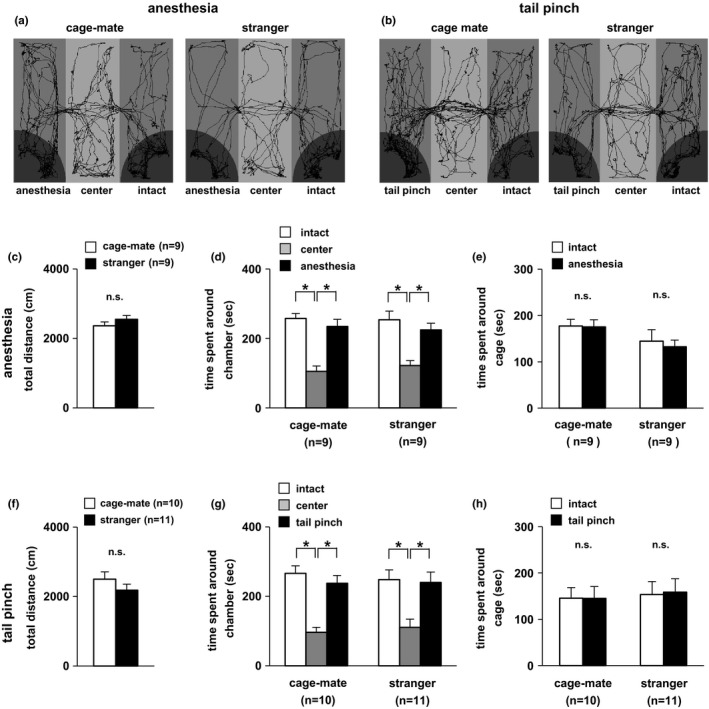
Preference tests for both anesthetized and tail‐pinched mice in the three‐chambered apparatus. (a) Sample trace of subject mice in the cage‐mate and stranger mouse conditions in the preference test for anesthetized and intact mice. (b) Sample trace of subject mice in the cage‐mate and stranger mouse conditions in the preference test for tail‐pinched mice. Preference tests for anesthetized and intact mice: total distance traveled (c), time spent in the chamber (d), and time spent around the cage (e). Preference tests for tail‐pinched mice and intact mice: total distance traveled (f), time spent in the chamber (g), and time spent around the cage (h). All data are presented as mean ± *SEM*. (c) *F*_1,16_ = 1.421, *p *=* *.2507. (d) Cage‐mate vs stranger: *F*_1,48_ = 0.0005, *p *=* *.9643; chamber: *F*_2,48_ = 33.202, *p* = .0001; cage‐mate vs stranger × chamber: *F*_2,48_ = 0.282, *p *=* *.7553. Cage‐mate: intact vs center, *t *=* *4.932, *p *=* *.00001; center vs anesthesia, *t *=* *5.792, *p *=* *.0001; intact vs anesthesia, *t *=* *0.861, *p *=* *.3937. Stranger: intact vs center, *t *=* *3.910, *p *=* *.0001; center vs anesthesia, *t *=* *5.030, *p *=* *.0001; intact vs anesthesia, *t *=* *1.120, *p *=* *.2682. (e) Cage‐mate vs stranger: *F*_1,32_ = 2.815, *p *=* *.1031; intact vs anesthesia: *F*_1,32_ = 0.096, *p *=* *.7590; cage‐mate vs stranger × intact vs anesthesia: *F*_1,32_ = 0.0003, *p *=* *.9568: cage‐mate, *p *=* *.147; stranger, *p *=* *.7043. (F) *F*_1,19_ = 1.371, *p *=* *.2562. (G) Cage‐mate vs stranger: *F*_1,57_ = 0.0001, *p *=* *.9872; chamber: *F*_2,57_ = 23.860, *p *=* *.0001; cage‐mate vs stranger × chamber: *F*_2,57_ = 0.228, *p *=* *.7972. Cage‐mate: intact vs center, *t *=* *4.826, *p *=* *.00001; center vs tail pinch, *t *=* *4.020, *p *=* *.0002; intact vs tail pinch, *t *=* *0.806, *p *=* *.4238. Stranger: intact vs center, *t *=* *4.096, *p *=* *.0001; center vs tail pinch, *t *=* *3.865, *p *=* *.0003; intact vs tail pinch, *t *=* *0.231, *p *=* *.8180. (H) Cage‐mate vs stranger: *F*_1,38_ = 0.160, *p *=* *.6912; intact vs tail pinch: *F*_1,38_ = 0.009, *p *=* *.9262; cage‐mate vs stranger × intact vs tail pinch: *F*_1,38_ = 0.009, *p *=* *.9243: cage‐mate, *F*_1,38_ = 0.0001, *p *=* *.9987; stranger, *F*_1,38_ = 0.018, *p *=* *.8944. Statistical significance is represented by asterisks: *p <* *.05

In this experiment, the degree of interest in the anesthetized cage‐mate mouse was examined. Subject mice were placed in a state where they could freely move between a wire cage with an intact and a wire cage with an anesthetized cage‐mate (Figure 1a,c–e). Subject mice spent almost the same time in the chamber of either side (Figure 1d). Consistently, there was no significant difference in the preference around the wire cage of either side (Figure 1e). Next, the same experiment was performed using stranger subject mice (non‐cage‐mates). Subject mice showed similar contact times for both chambers (Figure 1d). Subject mice spent an equivalent amount of time near both cages (Figure 1e). There were no significant differences in general activity such as total distance traveled between the cage‐mate and stranger mouse conditions (Figure 1c). Individual results showed there was no dominant trend in the time spent around each cage (Figure 3a). These results suggested that there was no difference in interest behavior toward cage‐mate or stranger mice and that there was a lack of preference for an anesthetized mouse over an intact mouse in both the stranger and cage‐mate mouse conditions.

### 3.2 Degree of interest in mice experiencing pain due to tail pinch

To examine the interest in cage‐mate mice experiencing pain, the same subject mice were subjected to this experiment. The tail‐pinch method was performed in cage‐mate mice (Figure 1b,f–h). No significant differences were found between the time spent in the chamber with the intact mouse and the time spent in the chamber with the tail‐pinched mouse (Figure 1g). Likewise, there were no significant differences between the time spent around the cage with the intact cage‐mate and the tail‐pinched cage‐mate mice (Figure 1h). Next, the same experiment was performed using stranger mice. There were no significant differences between the time spent in the chamber with the intact stranger mouse and the time spent in the chamber with the tail‐pinched stranger mouse (Figure 1g). Moreover, subject mice showed no significant differences in time spent around the two wire cages (Figure 1h). No significant difference was detected between the cage‐mate and stranger mouse conditions in distance traveled (Figure 1f). Individual results showed there was no dominant trend in the time spent around each cage (Figure 3b). These findings suggest that there was no difference in interest behavior toward cage‐mate and stranger mice and that there was a lack of preference for a tail‐pinched mouse over an intact mouse in both the stranger and cage‐mate mouse conditions.

### 3.3 Degree of interest in mice experiencing pain due to formalin injection

**Figure 2:**
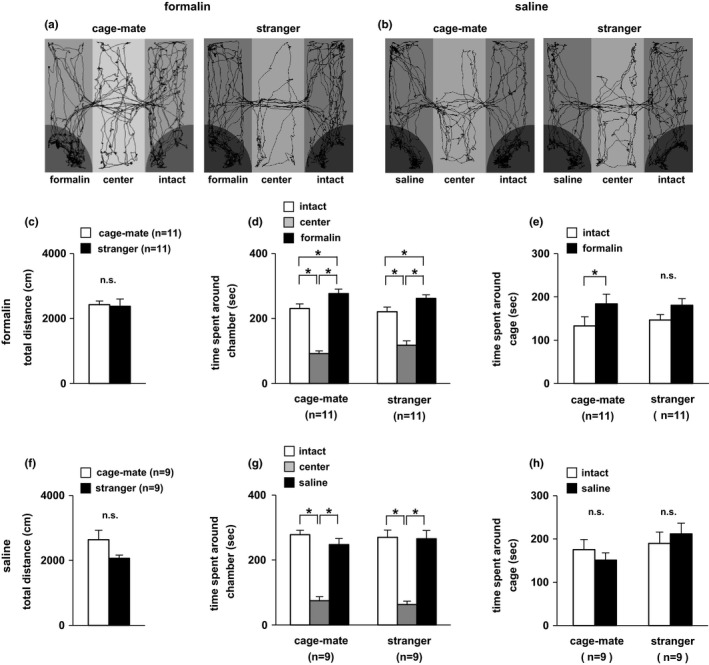
Preference tests of both formalin‐administered and saline‐administered mice in the three‐chambered apparatus. (a) Sample trace of subject mice in the cage‐mate and stranger mouse conditions in the preference test for formalin‐administered mice and intact mice. (b) Sample trace of subject mice in the cage‐mate and stranger mouse conditions in the preference test for formalin‐administered mice. Preference tests for saline‐administered and intact mice: total distance traveled (c), time spent in the chamber (d), and time spent around the cage (e). Preference tests for saline‐administered and intact mice: total distance traveled (f), time spent in the chamber (g), and time spent around the cage (H). All data are presented as mean ± *SEM*. (c) *F*_1,20_ = 0.034, *p *=* *.8555. (d) Cage‐mate vs stranger: *F*_1,60_ = 0.0001, *p *=* *.9938; chamber: *F*_2,60_ = 88.879, *p *=* *.0001; cage‐mate vs stranger × chamber: *F*_2,60_ = 1.464, *p *=* *.2395. Cage‐mate: intact vs center, *t *=* *10.214, *p *=* *.00001; center vs formalin, *t *=* *7.651, *p* = .0001; intact vs formalin, *t* = 2.563, *p* = .0128. Stranger: intact vs center, *t* = 7.984, *p* = .0001; center vs formalin, *t* = 5.723, *p* = .0001; intact vs formalin, *t* = 2.261, *p* = .0274. (e) Cage‐mate vs stranger: *F*_1,40_ = 0.004, *p* = .9510; intact vs formalin: *F*_1,40_ = 10.327, *p* = .0026; cage‐mate vs stranger × intact vs formalin: *F*_1,40_ = 0.650, *p* = .4249: cage‐mate, *F*_1,40_ = 8.079, *p* = .0070; stranger, *F*_1,40_ = 2.898, *p* = .0964. (f) *F*_1,16_ = 3.563, *p* = .0774. (g) Cage‐mate vs stranger: *F*_1,48_ = 0.002, *p* = .9641; chamber: *F*_2,48_ = 79.785, *p* = .0001; cage‐mate vs stranger × chamber: *F*_2,48_ = 0.427, *p* = .6548. Cage‐mate: intact vs center, *t* = 6.775, *p* = .00001; center vs saline, *t* = 7.970, *p* = .0001; intact vs saline, *t* = 1.195, *p* = .2377. Stranger: intact vs center, *t* = 7.979, *p* = .0001; center vs saline, *t* = 8.130, *p* = .0001; intact vs saline, *t* = 0.152, *p* = .8799. (H) Cage‐mate vs stranger: *F*_1,32_ = 2.672, *p* = .1119; intact vs saline: *F*_1,32_ = 0.003, *p* = .9598; cage‐mate vs stranger × intact vs saline: *F*_1,32_ = 0.996, *p* = .3259: cage‐mate, *F*_1,32_ = 3.465, *p* = .0719; stranger, *F*_1,32_ = 0.203, *p* = .6555. Statistical significance is represented by asterisks: *p <* *.05

**Figure 3:**
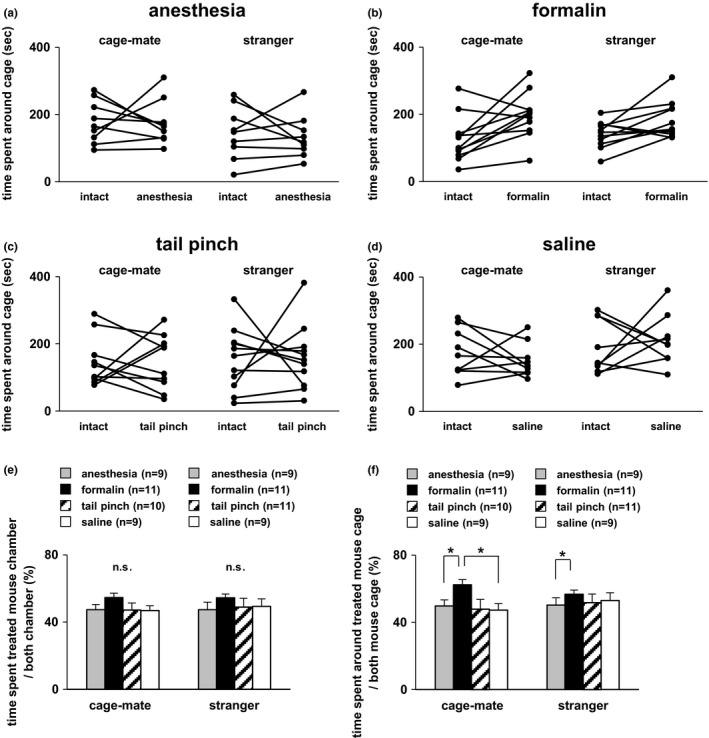
Empathic behavioral test for both cage‐mate mice and stranger mice. (a) Individual results of the empathic test for anesthetized and intact mice. (b) Individual results of the empathic test for formalin‐administered and intact mice. (c) Individual results of the empathic test for tail‐pinched and intact mice. (d) Individual results of the empathic test for saline‐administered and intact mice. (e) Mean time spent in the chamber with treated mice per time spent in both side chambers. (f) Mean time spent around the wire cage with treated mice per time spent around both side wire cages. All data are presented as mean ± *SEM*. (e) Cage‐mate vs stranger: *F*_1,71_ = 0.028, *p* = .8672; treatment: *F*_3,71_ = 1.489, *p* = .2249; cage‐mate vs stranger × treatment: *F*_3,71_ = 0.855, *p* = .4685. Cage‐mate: anesthesia vs formalin, *t* = 1.231, *p* = .2222; anesthesia vs tail pinch, *t* = 1.216, *p* = .2281; anesthesia vs saline, *t* = 0.370, *p* = .7126; formalin vs tail pinch, *t* = 0.012, *p* = .9906; formalin vs saline, *t* = 1.619, *p* = .1098; tail pinch vs saline, *t* = 1.595, *p* = .1151. Stranger: anesthesia vs formalin, *t* = 1.581, *p* = .1183; anesthesia vs tail pinch, *t* = 0.480, *p* = .6323; anesthesia vs saline, *t* = 1.036, *p* = .3038; formalin vs tail pinch, *t* = 1.160, *p* = .2499; formalin vs saline, *t* = 0.495, *p* = .6224; tail pinch vs saline, *t* = 0.606, *p* = .5465. (f) Cage‐mate vs stranger: *F*_1,71_ = 0.051, *p* = .8220; treatment: *F*_3,71_ = 2.992, *p* = .0366; cage‐mate vs stranger × treatment: *F*_3,71_ = 1.328, *p* = .2722. Cage‐mate: anesthesia vs formalin, *t* = 1.988, *p* = .050; anesthesia vs tail pinch, *t* = 0.898, *p* = .3720; anesthesia vs saline, *t* = 0.537, *p* = .5930; formalin vs tail pinch, *t* = 1.100, *p* = .2749; formalin vs saline, *t* = 2.551, *p* = .0129; tail pinch vs saline, *t* = 1.449, *p* = .1517. Stranger: anesthesia vs formalin, *t* = 2.101, *p* = .03923; anesthesia vs tail pinch, *t* = 0.512, *p* = .6105; anesthesia vs saline, *t* = 1.538, *p* = .1284; formalin vs tail pinch, *t* = 1.675, *p* = .0982; formalin vs saline, *t* = 0.488, *p* = .6273; tail pinch vs saline, *t* = 1.102, *p* = .2743. Statistical significance is represented by asterisks: *p <* *.05

Subsequently, to examine the interest in cage‐mate mice experiencing pain, we used cage‐mate mice experiencing pain in the paw caused by formalin injection. The formalin administration method was performed in cage‐mate mice (Figure 2a,c–e). Subject mice spent a significantly longer time in the chamber with a formalin‐administered cage‐mate mouse than in the chamber with the intact cage‐mate mouse (Figure 2d). Consistently, subject mice showed a preference for spending time around the wire cage with the formalin‐administered cage‐mate mouse (Figure 2e). Next, the same experiment was performed using stranger mice. Subject mice spent a longer time in the chamber with the formalin‐administered stranger mouse than in the chamber with the intact stranger mouse (Figure 2d). Subject mice showed no significant differences in time spent around the two wire cages (Figure 2e). Individual results showed that nine of 11 subject mice stayed longer around the wire cage with the formalin‐administered cage‐mate mouse (Figure 3b). No significant difference was detected between the cage‐mate and stranger mouse conditions in distance traveled (Figure 2c). Thus, these results suggest that the degree of interest toward formalin‐administered cage‐mate mice was enhanced.

### 3.4 Degree of interest in mice with swollen limbs due to saline injection

The same experiment was performed by injection of saline into the paw of mice. We confirmed the swelling limbs of the mice by due to rapid saline injection. We examined both cage‐mate and stranger mice. There were no significant differences between the time spent in the chamber with the saline‐injected mouse and intact mouse in the cage‐mate and stranger mouse conditions (Figure 2g). Likewise, no significant differences were found between the time spent around the cage with the saline‐injected mouse and the time spent around the opposite cage with the intact mouse in both the cage‐mate and stranger mouse conditions (Figure 2h). No significant difference was detected between the cage‐mate and stranger mouse conditions in distance traveled (Figure 2f). Individual results showed there was no dominant trend in the time spent around each cage (Figure 3d). These results indicate that subject mice were not interested in the state of incongruity and difficulty walking on the paw of the cage‐mate or stranger mice.

### 3.5 Degree of interest in mice experiencing pain in an opaque cylinder

**Figure 4:**
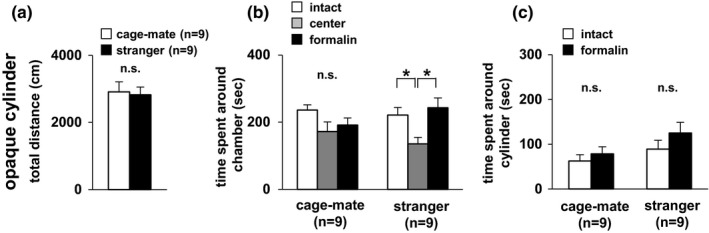
Empathic behavioral test for both cage‐mate and stranger mice in the opaque cylinder. Preference tests for formalin‐administered and intact mice in the opaque cylinder: total distance traveled (a), time spent in the chamber (b), and time spent around the cylinder (c). All data are presented as mean ± *SEM*. (a) *F*_1,16_ = 0.053, *p* = .8200. (b) Cage‐mate vs stranger: *F*_1,48_ = 0.0001, *p* = 1.0; chamber: *F*_2,48_ = 5.963, *p* = .0049; cage‐mate vs stranger × chamber: *F*_2,48_ = 1.936, *p* = .1554. Cage‐mate: intact vs center, *t *=* *1.951, *p *=* *.0569; center vs formalin, *t *=* *0.580, *p *=* *.5647; intact vs formalin, *t *=* *1.371, *p *=* *.1767. Stranger: intact vs center, *t *=* *2.591, *p *=* *.0126; center vs formalin, *t *=* *3.245, *p *=* *.0021; intact vs formalin, *t *=* *0.654, *p *=* *.5163. (c) Cage‐mate vs stranger: *F*_1,32_ = 3.863, *p *=* *.0581; intact vs formalin: *F*_1,32_ = 1.933, *p *=* *.1740; cage‐mate vs stranger × intact vs formalin: *F*_1,32_ = 0.294, *p *=* *.5913: cage‐mate, *F*_1,32_ = 1.013, *p* = .3218; stranger, *F*_1,32_ = 3.145, *p* = .0857. Statistical significance is represented by asterisks: *p <* *.05

Blocking visual information, we conducted an experiment with mice with spontaneous pain in the paw caused by formalin injection. Formalin‐injected mice and untreated mice were placed in opaque cylinders instead of the wire cages, which were placed at the corners of the chamber. Subject mice showed no significant differences in time spent around the three chambers (Figure 4b). In contrast, subject mice spent similar time in both chambers when they contained stranger mice (Figure 4b). There was no difference between time spent around the opaque cylinder containing the formalin‐administered mouse and time spent around the opaque cylinder containing the intact mouse in both the cage‐mate and stranger mouse conditions (Figure 4c). There was no significant difference in distance traveled between the cage‐mate and stranger mouse conditions (Figure 4a). These results suggest that without visual information, the subject mice do not show preference for the cage‐mate mouse in the pain state.

### 3.6 Degree of interest in the scent of mice experiencing pain

**Figure 5:**
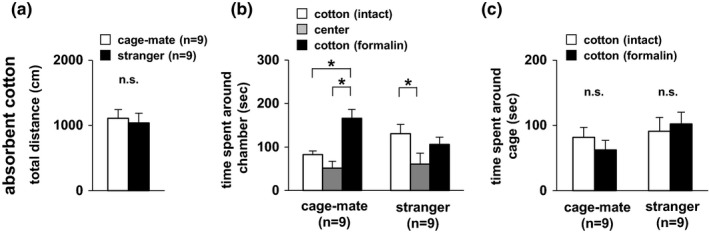
Empathic behavioral test for both the absorbent cotton with the scent of the formalin‐administered mouse and the absorbent cotton with the scent of an intact mouse. Preference tests for the absorbent cotton with the scent of the formalin‐administered mouse and for the absorbent cotton with the scent of an intact mouse in the wire cage: total distance traveled (a), time spent in the chamber (b), and time spent around the cage (c). All data are presented as mean ± *SEM*. (a) *F*_1,16_ = 0.123, *p* = .7304. (b) Cage‐mate vs stranger: *F*_1,48_ = 0.003, *p* = .9565; chamber: *F*_2,48_ = 9.333, *p* = .0004; cage‐mate vs stranger × chamber: *F*_2,48_ = 4.299, *p* = .0192. Cage‐mate: intact vs center, *t* = 1.166, *p* = .2493; center vs formalin, *t* = 4.335, *p* = .0001; intact vs formalin, *t* = 3.169, *p* = .0026. Stranger: intact vs center, *t* = 2.632, *p* = .0113; center vs formalin, *t* = 1.709, *p* = .0939; intact vs formalin, *t* = 0.923, *p* = .3605. (C) Cage‐mate vs stranger: *F*_1,32_ = 1.981, *p* = .1689; intact vs formalin: *F*_1,32_ = 0.050, *p* = .8244; cage‐mate vs stranger × intact vs formalin: *F*_1,32_ = 0.755, *p* = .3914: cage‐mate, *F*_1,32_ = 0.145, *p* = .7058; stranger, *F*_1,32_ = 2.591, *p* = .1173. Statistical significance is represented by asterisks: *p <* *.05

Next, we examined whether subject mice were interested in odor information. Both an absorbent cotton with the scent of the formalin‐administered mouse and an absorbent cotton with the scent of an intact mouse were prepared. These two types of absorbent cotton were placed in wire cages at the corner of the chamber. Subject mice spent a significantly longer time in the chamber with the scent of the formalin‐administered cage‐mate mouse than in the chamber with the scent of the intact cage‐mate mouse (Figure 5b). Subject mice spent a significantly longer time in the chamber with the scent of the intact stranger mouse than in the chamber with the scent of the formalin‐administered stranger mouse (Figure 5b). However, there was no difference between the time spent around the wire cage containing the absorbent cotton with the intact mouse scent and the time spent around the wire cage containing the absorbent cotton with the formalin‐administered mouse scent in both the cage‐mate and stranger mouse conditions (Figure 5c). These results suggested that the subject mice were interested in odor information of the formalin‐administered cage‐mate mouse.

## 4 DISCUSSION

In this study, mice showed no heightened interest in anesthetized conspecifics or conspecifics with swollen limbs, but showed interest in formalin‐injected conspecifics. Moreover, test mice also showed similar responses to formalin‐injected cage‐mates or strangers.

Similar results showing social preference toward cage‐mates who received formalin injection have been previously reported (Watanabe, 2012). Furthermore, we showed that mice also show social preference toward stranger mice. Although the social preference response toward stranger mice is lower than that toward cage‐mate mice, it is suggested that important information could be obtained from stranger formalin‐injected mice, urging action. It has been demonstrated that rodents recognize the pain of conspecifics and show emotional responses (Preston & de Waal, 2002). It is suggested that the results obtained in this study signify empathic behavior, which is part of the emotional response in mice.

The type of information obtained from conspecifics experiencing pain that leads to empathic behavior remains unclear. However, it has been shown that when visual information is blocked, the emotional transmission of pain between mice is inhibited (Langford et al., 2006). Visual information is considered to be necessary. We also demonstrated that when visual information is blocked, mice no longer showed preference for conspecifics experiencing pain. Animals have the ability to look at the movement of others and identify their state. Quail can identify the state of others injected with methamphetamine or ketamine by observing their behavior (Yamazaki, Shinohara, & Watanabe, 2004). Mice show interest in cage‐mates that exhibit abnormal behavior (Yang et al., 2016). In this study, mice showed lack of social preference toward individuals injected with physiological saline solution, which caused paw swelling. To wit, it is suggested that no attention signal was transmitted from conspecifics who were not in a pain state even though their legs were swollen. From the above, the social approach behaviors toward conspecifics experiencing pain suggest that mice discriminate among the sensory states of conspecifics (Langford et al., 2006).

Furthermore, olfactory cues are also reportedly important for empathic behavior (Smith, Hostetler, Heinricher, & Ryabinin, 2016). Mice most often use olfactory cues rather than other sensory cues in individual identification (Corridi, Chiarotti, Bigi, & Alleva, 1993). In this study, empathic behavior was not observed when enclosing the mouse experiencing pain in an opaque cylinder. However, test mice showed interest in absorbent cotton imbued with the odor of mice experiencing pain. To wit, the results of this study are consistent with previous studies, suggesting that both visual and olfactory cues are essential for empathic behavior in mice.

In this experiment, the subject mice did not show social preference toward conspecifics who were in pain due to tail pinch. The clothespins were placed in sight of the mouse experiencing pain due to tail pinch. The mouse experiencing pain due to tail pinch is presumed to have been agitated by the clothespins; we observed that the tail‐pinched mice frequently attempted to bite both the clothespin and the wire cage. Therefore, it is speculated that the subject mouse, who was watching this situation, avoided approaching the conspecific because of the aggressive behavior.

Mice did not show social preference for anesthetized conspecifics. The subject mouse may have interpreted the abnormal sleeping of their conspecific as a normal sleeping state. In nature, mice rarely have the opportunity to see stunned conspecifics, so mice may not have the ability to recognize it as abnormal.

It was shown that mouse empathic behavior differed among five mouse strains (Keum & Shin, 2016). Moreover, the social behavior of mice varied depending on their strain (Drapeau, Dorr, Elder, & Buxbaum, 2014). These reports indicate that some genetic factors may be related to empathic behavior. As mice witness cage‐mate mice experiencing pain, they become more susceptible to pain (Langford et al., 2006). Rats try to rescue cage‐mate rats when they are in a distressed environment (Sato, Tan, Tate, & Okada, 2015; Silberberg, Allouch, Sandfort, Kearns, & Karpel, 2014). Rats avoid listening to screams of conspecifics (Otsuka, Yanagi, & Watanabe, 2009). Furthermore, when both rats and pigeons see that their cage‐mates were in a state of pain induced by electric shock, operant learning was inhibited (Church, 1959; Watanabe & Ono, 1986). This indicates that witnessing conspecifics experiencing distress causes discomfort to the observer; by helping the conspecific escape the source of distress, the unpleasant behavior is reduced, and presumably the aversive stimulus is avoided (Watanabe, 2011). The representation of discomfort of others is often a signal of danger to oneself, having aversion toward functions as a crisis avoidant. In other words, reducing the discomfort of conspecifics results in reducing the aversive state.

In experiments using rodents, many studies have reported that cage‐mates are the only ones that show empathetic behavior (Leiberg & Anders, 2006; Panksepp et al., 2007; de Vignemont & Singer, 2006). It is unclear why no emotional contagion and empathic behavior are noted between stranger conspecifics although they share the same movement and shape. Generally, interest in stranger mice is higher than in cage‐mate mice, as more information may be elicited from stranger mice (Crawley, 2004). In human society, being unable to recognize others’ emotions would interfere with communication. Showing similar attention to stranger and cage‐mate mice who are experiencing pain is considered to represent empathy‐like behavior.

In ASD, schizophrenia, personality disorders, and depression, impairments in empathic behavior are observed (Bernhardt & Singer, 2012; Bird et al., 2010; Bora et al., 2009). Especially in ASD, both cognition and empathy of emotion are important in both diagnosis and treatment. In 11 strains of mice, there was no association between social novelty preference and empathy for fear (Keum et al., 2016). These reports indicate that sociality and empathy are controlled by different mechanisms, and testing empathy is not identical to testing sociality in neuropsychiatric model mice. Investigating the mechanisms of empathic behavior in rodents is important for understanding the underlying mechanisms involved in human neuropsychiatric disorders and for contributing to the development of new therapeutic targets for these diseases.

There is no established precise method for testing empathy in mice (Langford et al., 2006; Wahlsten, 2010). If this method is established, empathic behavior can be easily investigated using neuropsychiatric disorder model mice. The present study conducted experiments using the widely used three‐chambered sociability test equipment. Therefore, our method is suggested as a straightforward way of testing empathic behavior in mice.

This study suggests that mice show empathic behavior. Both visual information and olfactory information are important for this. It would be interesting to investigate how the subject mice would react to their conspecifics experiencing distress, if they were allowed to approach them. Further research is needed to this end. In conclusion, this experimental method is suitable for the investigation of empathic behavior in mice.

## CONFLICT OF INTEREST

The authors declare that they have no competing financial interests.

## AUTHOR CONTRIBUTIONS

All authors had full access to all the data in the study and take full responsibility for the integrity of the data and the accuracy of the data analysis. H.U., M.O., and T.I. conceived and designed the study. H.U. and S.S. acquired, analyzed, and interpreted the data and carried out statistical analysis. H.U. and M.O. drafted the manuscript. S.M., N.K., K.W., Y.M., S.A., and T.I. critically revised the manuscript for important intellectual content. M.O., S.A., and T.I. supervised the study.
